# Does Antiplatelet Therapy during Bridging Thrombolysis Increase Rates of Intracerebral Hemorrhage in Stroke Patients?

**DOI:** 10.1371/journal.pone.0170045

**Published:** 2017-01-17

**Authors:** Anne Broeg-Morvay, Pasquale Mordasini, Agnieszka Slezak, Kai Liesirova, Julia Meisterernst, Gerhard Schroth, Marcel Arnold, Simon Jung, Heinrich P. Mattle, Jan Gralla, Urs Fischer

**Affiliations:** 1 Department of Neurology, Inselspital, University Hospital Bern and University of Bern, Bern, Switzerland; 2 Department of Diagnostic and Interventional Neuroradiology, Inselspital, University Hospital Bern and University of Bern, Bern, Switzerland; Medical University Innsbruck, AUSTRIA

## Abstract

**Background:**

Symptomatic intracerebral hemorrhage (sICH) after bridging thrombolysis for acute ischemic stroke is a devastating complication. We aimed to assess whether the additional administration of aspirin during endovascular intervention increases bleeding rates.

**Methods:**

We retrospectively compared bleeding complications and outcome in stroke patients who received bridging thrombolysis with (tPA+ASA) and without (tPA-ASA) aspirin during endovascular intervention between November 2008 and March 2014. Furthermore, we analyzed bleeding complications and outcome in antiplatelet naïve patients with those with prior or acute antiplatelet therapy.

**Results:**

Baseline characteristics, previous medication, and dosage of rtPA did not differ between 50 tPA+ASA (39 aspirin naïve, 11 preloaded) and 181 tPA-ASA patients (p>0.05). tPA+ASA patients had more often internal carotid artery (ICA) occlusion (p<0.001), large artery disease (p<0.001) and received more often acute stenting of the ICA (p<0.001). 10/180 (5.6%) tPA-ASA patients and 3/49 (6.1%) tPA+ASA patients suffered a sICH (p = 1.0). Rates of asymptomatic intracerebral hemorrhage, systemic bleeding complications and outcome did not differ between both groups (p>0.1). There were no differences in bleeding complications and mortality among 112 bridging patients with antiplatelet therapy (62 preloaded, 39 acute administration, 11 both) and 117 antiplatelet naïve patients. In a logistic regression analysis, aspirin administration during endovascular procedure was not a predictor of sICH.

**Conclusion:**

Antiplatelet therapy before or during bridging thrombolysis in patients with acute ischemic stroke did not increase the risk of bleeding complications and had no impact on outcome. This finding has to be confirmed in larger studies.

## Introduction

Early administration of aspirin after intravenous thrombolysis (IVT) in patients with acute ischemic stroke increases the risk of symptomatic intracerebral hemorrhage (sICH) and does not improve outcome [1]. Current stroke guidelines and study protocols do not recommend the use of antiplatelets or anticoagulants in the first 24 hours after treatment with intravenous recombinant tissue plasminogen activator (iv rt-PA). [2–5]

IVT followed by endovascular therapy (EVT) is effective in stroke patients with proximal vessel occlusions [6, 7]. In patients with acute occlusions or relevant stenosis of large extracranial vessels acute stenting is sometimes necessary [8, 9]. Administration of antiplatelets is required to prevent reocclusion of stents [10, 11]. Furthermore, EVT can cause endothelial damage resulting in vessel stenosis, dissections and reocclusions [12, 13]. Antiplatelets might prevent thrombus formation and vessel reocclusion in damaged vessel. However it remains unclear, whether the potential benefit of antiplatelets during bridging thrombolysis is outweighed by bleeding complications.

Based on the current literature we hypothesized an increase in bleeding complications in bridging patients who receive additional antiplatelet therapy during endovascular intervention. We therefore assessed rates of sICH, asymptomatic intracerebral hemorrhage (aICH), systemic bleeding complications and outcome in patients with bridging thrombolysis with (tPA+ASA) and without (tPA-ASA) additional aspirin administration during endovascular intervention.

## Materials and Methods

### Patients

This study is based on the Bernese stroke registry, a prospective data collection of patients with acute ischemic stroke. All patients who received combined IVT and EVT (bridging therapy) were included in this retrospective analysis. Demographic data, previous medication, vascular risk factors, laboratory findings, treatment modality, and time from symptom onset to treatment were recorded. Clinical evaluation was performed by a stroke neurologist immediately after admission in the emergency room using the National Institute of Health Stroke Scale (NIHSS) score. Stroke etiology was classified according to Trial of ORG 10172 in Acute Stroke Treatment (TOAST) criteria [14].

Thrombolysis in our institution is performed according to international and institutional guidelines. The general approach at our centre has been described previously [9, 15, 16]. Immediately after clinical evaluation all patients underwent CT or MR imaging to rule out intracranial hemorrhage and CT or MR angiography (MRA) to assess vessel occlusion and if present its location. Intravenous thrombolysis is directly started in the scanner. Patients with proximal vessel occlusions were considered as candidates for endovascular therapy. Endovascular therapy was performed with the consent of the patient or his family immediately after CT or MRI if: 1) diagnosis of ischemic stroke was established; 2) baseline NIHSS score was ≥ 4 points or isolated aphasia or hemianopia was present; 3) hemorrhage on cranial CT or MRI was excluded; 4) vessel occlusion correlated with the neurological deficit; 5) symptom duration was not longer than 24 hours; and 6) no individual clinical or premorbid conditions or laboratory findings advised against thrombolysis [16]. Digital subtraction angiography (DSA) was performed via a transfemoral approach using a biplane, high-resolution angiography system. In general, a four vessel cerebral angiography was performed prior to the intervention. Endovascular recanalization procedures consisted of a combination of several approaches depending on occlusion pattern and operator preference: intra-arterial thrombolysis using urokinase, mechanical recanalization using direct thrombaspiration through a catheter placed proximal to the thrombus and stent retrievers retracting the thrombus after stent expansion within in the thrombus area and placement of extracranial stents with or without pre- or postdilatation. The interventional neuroradiologists decided together with the stroke neurologist on the use of urokinase, mechanical intervention and—if necessary—stenting of the extracranial internal carotid artery (ICA). In general, patients received aspirin intravenously during or immediately after the endovascular intervention (ASA dosage: mean 341 mg, median 300mg) if stenting was performed, unless they were already under a treatment with antiplatelets. Dual antiplatelet therapy was not administrated within the first 24 hours after symptom onset. 24 hours after treatment, or in any case of clinical deterioration a CT or MRI scan was performed. sICH and aICH were classified according to the PROACT II study protocol [17]. Furthermore, any systemic bleeding occurrence was documented (excluding local hematoma at the catheter incision site). Recanalization rates were assessed immediately after angiography using the thrombolysis in myocardial infarction (TIMI) classification. [18] Furthermore, status of vessel recanalization was assessed in patients who underwent CT or MR angiography after 24 hours. In patients with extracranial stenting, information on stent patency was obtained by ultrasound and/or CT angiography. Clinical outcome was prospectively assessed 3-months after the stroke using the modified Rankin scale (mRS). The study was performed according to the ethical guidelines of the Canton of Bern and with corresponding permission (Kantonale Ethikkommission Bern, Hörsaaltrakt Pathologie, Eingang 43A, Büro H372, Murtenstrasse 31, 3010 Bern).

### Statistical Analysis

Statistical analysis was performed using SPSS 21 (SPSS Inc., Chicago, Illinois, USA). We compared baseline characteristics, risk factors, stroke etiology, laboratory findings, blood pressure, site of vessel occlusion, therapy and outcome between tPA+ASA and tPA-ASA patients. Differences between the two groups were assessed using Fisher’s exact test comparison for categorical variables and Mann Whitney test for comparison of continuous variables. To detect the influence of any antiplatelet therapy in acute stroke patients who underwent bridging thrombolysis, we compared bleeding complications and outcome in antiplatelet naïve patients versus those with previous or acute antiplatelet therapy. Complete recanalization was defined as TIMI 3. Outcome was dichotomized into favorable (mRS 0–2) and poor clinical outcome (mRS 3–6). Logistic regression analysis was used to determine the predictors of sICH and aICH. We included all variables with a p value <0.2 in the univariate analysis. We analyzed whether aspirin in combination with aforementioned relevant variables had an impact on sICH and aICH. A two-sided p value <0.05 was considered statistically significant.

## Results

From 1^st^ November 2008 to 31^st^ March 2014 1145 patients received thrombolysis for acute ischemic stroke. 231 underwent bridging therapy and 50 of these received aspirin intravenously in addition to rtPA (39 antiplatelet naïve, 11 preloaded). Baseline characteristics and vascular risk factors of tPA-ASA and tPA+ASA patients are shown in Table 1.

**Table 1:** Baseline characteristics and vascular risk factors.

	Bridging without ASA (n = 181)	Bridging with ASA (n = 50)	p
Sex female, n (%)	82 (45.3)	13 (26.0)	0.015
Age, mean (SD)	69 (14)	66 (13)	0.049
NIHSS score, median (range)	15 (2–37)	15 (3–36)	0.493
**Vascular risk factors**			
Diabetes mellitus, n (%)	29/180 (16.1)	12 (24.0)	0.213
Hypertension, n (%)	111/180 (61.7)	34 (68.0)	0.508
Hypercholesterolemia, n (%)	94/179 (52.5)	23/49 (46.9)	0.522
Current smoking, n (%)	36/158 (22.8)	10/44 (22.7)	1.0
**Etiology**			
TOAST			
large artery atherosclerosis	15/178 (8.4)	17 (34.0)	<0.001
cardioembolism	90/178 (50.6)	8 (16.0)	<0.001
small-vessel occlusion	0	0	-
other determined etiology	0	6 (12.0)	<0.001
undetermined etiology	73/178 (41.0)	19 (38.0)	0.746
Atrial fibrillation, n (%)	74/153 (48.4)	9/43 (20.9)	0.001
Coronary heart disease, n (%)	31/180 (17.2)	6 (12.0)	0.514
**Laboratory findings**			
INR, mean (SD)	1.06 (0.1)	1.04 (0.8)	0.179
Platelet count, mean (SD)	214 (65)	231 (72)	0.112
Serum glucose, mmol/l, mean (SD)	7.1 (2.1)	7.4 (2.4)	0.250
**Blood pressure**			
Systolic blood pressure on admission, mean (SD)	153 (29)	160 (23)	0.062
Diastolic blood pressure on admission, mean (SD)	82 (18)	88 (20)	0.130
Maximum systolic blood pressure during intervention, mean (SD)	172 (25)	175 (24)	0.436
Maximum diastolic blood pressure during intervention, mean (SD)	85 (18)	91 (18)	0.062
**Anterior circulation stroke, n (%)**	156 (86.2)	41 (82.0)	
ICA	43 (23.8)	30 (60.0)	<0.001
M1/2	113 (62.4)	11 (22.0)	<0.001
**Posterior circulation stroke, n (%)**	25 (13.8)	9 (18.0)	
BA	23 (12.7)	9 (18.0)	0.357
other	2 (1.1)	0	-

**Abbreviations:** ASA: Acetyl salicylic acid, SD: standard deviation, NIHSS: National Institutes of Health Stroke Scale, TOAST: Trial of ORG 10172 in Acute Stroke Treatment, INR: international normalized ratio, ICA: internal carotid artery, M1/2: Segment 1 and 2 of the middle cerebral artery, BA: Basilar artery

**Fig 1:**
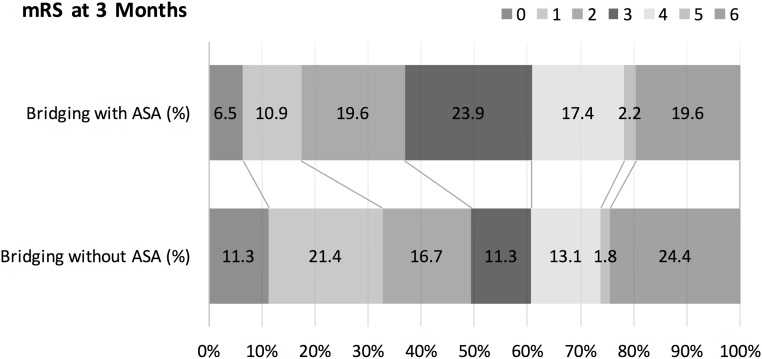
Modified Rankin Scale at 3 months.

Therapeutic approaches in both groups are shown in Table 2. Stroke severity (NIHSS score), risk factors, laboratory findings, blood pressure, time to treatment, preexisting antiplatelet therapy, and dosage of thrombolytics did not differ between both groups (p>0.05). tPA+ASA patients were more often men (p = 0.015), were older (p = 0.049), had more often large artery disease (p<0.001), atrial fibrillation (p = 0.001), ICA occlusions (p<0.001) and received more often acute stenting of the ICA (p<0.001). Clinical and radiological outcome is provided in Table 3 and Fig 1. Complete recanalization rate did not differ between tPA+ASA and tPA-ASA patients (p>0.1). Rates of sICH and aICH, systemic bleeding complications and outcome did not differ between tPA+ASA and tPA-ASA patients (p>0.1). In a logistic regression model, aspirin administration during endovascular procedure was not a predictor of sICH and aICH (p>0.05). Diabetes (p<0.038) and NIHSS score (p = 0.036) were independent predictors of sICH.

**Table 2:** Therapy.

	Bridging without ASA (n = 181)	Bridging with ASA (n = 50)	p
**Any preexisting antiplatelets, n (%)**	62/179 (34.6)	11 (22.0)	0.122
Aspirin, n (%)	48/62 (77.4)	10/11 (90.9)	
Clopidogrel, n (%)	13/62 (21.0)	0	
Dual Antiplatelet therapy, n (%)	1/62 (1.6)	1/11 (9.1)	
Unclear	2	0	
**Bridging Thrombolysis**			
**IVT**			
Minutes from onset to start of IVT, mean (range)	164 (58)	166 (58)	0.654
rtPA dose (mg), mean (SD)	51.5 (14.0)	54.2 (14.1)	0.242
**ET**			
Minutes from onset to start of ET, mean (range)	270 (83)	297 (102)	0.102
Mechanical thrombolysis, n (%)	165 (91.2)	47 (94.0)	0.771
Stenting intervention, n (%)	8 (4.4)	36 (72.0)	<0.001
Intracranial stenting, n (%)	3 (1.7)	9 (18.0)	<0.001
Extracranial stenting, n (%)	5 (2.8)	32 (64.0)	<0.001
Administration of Urokinase, n (%)	51 (28.2)	18 (36.0)	0.298
Combined Urokinase and mechanical thrombolysis, n (%)	35 (68.6)	15 (83.3)	0.358
Combined Urokinase and Stenting, n (%)	3 (5.9)	11 (61.1)	<0.001
Urokinase dose (IU), mean (SD)	123000 (238000)	154000 (261000)	0.344

**Abbreviations:** ASA: Acetyl salicylic acid, IVT: intravenous thrombolysis, ET endovascular thrombectomy, SD: standard deviation

**Table 3:** Outcome.

	Bridging without ASA (n = 181)	Bridging with ASA (n = 50)	p
**Complete vessel recanalization (TIMI 3)**			
immediately after endovascular intervention	116/179 (64.8)	30 (60.0)	0.618
24 h after endovascular intervention	106/137 (77.4)	25/39 (64.1)	0.101
**Complete vessel recanalization (only patients with extracranial stents)**			
24 h after endovascular stenting	7/8 (87.5)*	31/36 (86.1)	1.0
**Bleeding complications**			
sICH, n (%)	10/180 (5.6)	3/49 (6.1)	1.0
aICH, n (%)	37/180 (20.6)	9/48 (18.8)	0.843
systemic bleeding, n (%)	8 (4.4)	1 (2.0)	0.688
**Clinical outcome**			
favourable outcome (mRS 0–2) after 3 months, n (%)	83/168 (49.4)	17/46 (37.0)	0.182
mortality	41/168 (24.4)	9/46 (19.6)	0.560

**Abbreviations:** ASA: Acetyl salicylic acid, TIMI: thrombolysis in myocardial infarction, sICH: symptomatic intracerebral hemorrhage, aICH: asymptomatic intracerebral hemorrhage, mRS: modified Rankin Scale.

*5 out of 8 patients had preexisting antiplatelet therapy (including the patient who did not recanalize completely)

We analyzed bleeding complications and outcome in antiplatelet naïve patients with those with previous or acute antiplatelet therapy (Table 4). Patients with previous antiplatelet therapy were older (p = 0.05), more often men (p = 0.031), had a higher NIHSS score (p = 0.008), had more often diabetes (p = 0.014), hypertension (p = 0.004) and coronary heart disease (p<0.001). Furthermore, they had more often an occlusion of the ICA (p = 0.023) and were more often treated with extracranial stents (p<0.001). There were no differences in bleeding complications and mortality among 112 bridging patients with previous or acute antiplatelet therapy (62 preloaded, 39 acute administration, 11 both) and 117 patients who were antiplatelet naïve (information on antiplatelet premedication missing in 2 patients). However, outcome at 3 months was more favorable in antiplatelet naïve patients than in patients with antiplatelets (p = 0.014).

**Table 4:** Comparison between antiplatelet naïve patients and patients with prior and/or acute antiplatelet therapy^†^.

	Bridging without any Antiplatelets (n = 117)	Bridging with any Antiplatelets (n = 112)	p
***Baseline***			
Sex female, n (%)	56 (47.9)	37 (33.0)	0.031
Age, mean (SD)	66 (16)	71 (12)	0.05
NIHSS score, median (range)	14 (8)	17 (7)	0.008
**Vascular risk factors**			
Diabetes mellitus, n (%)	13/116 (11.2)	27 (24.1)	0.014
Hypertension, n (%)	63/116 (54.3)	82 (73.2)	0.004
Hypercholesterolemia, n (%)	55/115 (47.8)	62/111 (55.9)	0.234
Current smoking, n (%)	27/106 (25.5)	19/94 (20.2)	0.404
**Etiology**			
Atrial fibrillation, n (%)	44/96 (45.8)	39/100 (39.0)	0.386
Coronary heart disease, n (%)	7/116 (6.0)	30 (26.8)	<0.001
**Laboratory findings**			
INR, mean (SD)	1.05 (0.1)	1.05 (0.1)	0.315
Platelet count, mean (SD)	217 (64)	219 (71)	0.728
Serum glucose, mmol/l, mean (SD)	7.0 (2.1)	7.4 (2.2)	0.121
**Blood pressure**			
Systolic blood pressure on admission, mean (SD)	155 (31)	154 (24)	0.827
Diastolic blood pressure on admission, mean (SD)	83 (18)	83 (20)	0.669
Maximum systolic blood pressure during intervention, mean (SD)	173 (26)	173 (23)	0.639
Maximum diastolic blood pressure during intervention, mean (SD)	86 (17)	87 (19)	0.96
**Anterior circulation stroke, n (%)**	100 (85.5)	96 (85.7)	1.000
ICA	29 (24.8)	44 (39.3)	0.023
M1/2	71 (60.7)	52 (46.4)	0.034
**Posterior circulation stroke, n (%)**			
BA	15 (12.8)	16 (14.3)	0.847
other	2 (1.7)	0	-
***Therapy***			
Any preexisting antiplatelets, n (%)	0	73 (65.2)	
Additional ASA during bridging, n (%)	0	50 (44.6)	
**Bridging Thrombolysis**			
**IVT**			
Minutes from onset to start of IVT,mean (range)	162 (52)	167 (64)	0.732
rtPA dose, mg iv, mean (SD)	51.9 (13.6)	52.3 (14.7)	0.497
**ET**			
Minutes from onset to start of ET, mean (range)	272 (80)	278 (94)	0.497
Administration of Urokinase ia, n (%)	32 (27.4)	37 (33.0)	0.389
Urokinase dose, IU ia, mean (SD)	127000 (255000)	135000 (232000)	0.463
Mechanical thrombolysis, n (%)	107 (91.5)	103 (92.0)	1.0
Stenting, n (%)	3 (2.6)	41 (36.6)	<0.001
***Outcome***			
**Complete vessel recanalization (TIMI 3)**			
immediately after endovascular intervention	77/115 (67.0)	68 (60.7)	0.337
24 h after endovascular intervention	70/94 (74.5)	60/80 (75.0)	1.0
**Bleeding complications**			
sICH, n (%)	7 (6.0)	6/110 (5.5)	1.0
aICH, n (%)	22 (18.8)	23/109 (21.1)	0.740
systemic bleeding, n (%)	3 (2.6)	6 (5.4)	0.325
**Clinical outcome**			
favourable outcome (mRS 0–2) after 3 months, n (%)	60/108 (55.6)	40/104 (38.5)	0.014
mortality	23/108 (21.3)	26/104 (25.0)	0.625

**Abbreviations:** SD: standard deviation, NIHSS: National Institutes of Health Stroke Scale, INR: international normalized ratio, ICA: internal carotid artery, M1/2: Segment 1 and 2 of the middle cerebral artery, BA: Basilar artery, ASA: Acetyl salicylic acid, IVT: intravenous thrombolysis, ET endovascular thrombectomy, TIMI: thrombolysis in myocardial infarction, sICH: symptomatic intracerebral hemorrhage, aICH: asymptomatic intracerebral hemorrhage, mRS: modified Rankin Scale

^†^data about antiplatelet premedication missing in 2 patients of whom 1 patient had an aICH

## Discussion

This monocenter study of 231 stroke comparing patients with and without aspirin administration during bridging thrombolysis has three main findings: 1) There were neither an increase in sICH or aICH nor in any systemic bleeding complications, neither in univariate analysis nor in a regression model. 2) Outcome at three months did not differ among both groups. 3) Even when comparing antiplatelet naïve patients with those receiving antiplatelets prior or during endovascular intervention there was no difference in bleeding complications and mortality.

Our findings are in contrast to our hypothesis and the current literature. The ARTIS trial investigated whether infusion of 300 mg aspirin started within 90 minutes of intravenous thrombolysis with alteplase resulted in improved functional outcome in 642 patients. [1, 19]. The trial was stopped early because of an increased rate of sICH in the combination group (4% versus 2%, p = 0.04) with no improvement in outcome. Furthermore, data of the SITS-ISTR showed an absolute 1.4% increase of sICH in patients with previous use of antiplatelet therapy with no clear effect on outcome. [20]. In contrast to these studies we could not detect an increase in rates of sICH, aICH or any bleeding complications, neither in patients receiving aspirin acutely nor in patients pretreated with antiplatelets. However, compared to the ARTIS trial aspirin in our study was started more than 90 min after intravenous thrombolysis, which might partly explain the difference of our results compared to ARTIS and should therefore be considered in future study protocols. Rates of sICH were in line with the current literature on bleeding complications after bridging thrombolysis [6].

The rationale behind early administration of aspirin in patients receiving thrombolysis in previous studies is the prevention of reocclusion after early recanalization. [1]. Early reocclusion after initial recanalization occurs in 14–34% of patients and is associated with clinical deterioration and poor functional outcome [21–23]. In our study the main reason for aspirin administration during endovascular procedure was the prevention of acute reocclusion of extra- and/or intracranial stents. There are no randomized trials assessing the risk and benefit of antiplatelets for stenting of acute occlusions of extracranial vessels in patients with bridging thrombolysis. According to a consensus document on carotid stenting, patients should be pretreated with dual antiplatelet therapy [24]. The consensus document is mainly based on elective stenting of extracranial vessels [25–28] and on the evidence of major acute coronary trials, where early reocclusion of stented vessels is a major challenge [29]. In our study early recanalization rates between tPA+ASA and tPA-ASA patients did not differ and more than 80% of patients with acute stenting of extracranial vessels had a complete recanalization after 24 hours. However, our data are underpowered to assess the impact of antiplatelet therapy on recanalization rates after bridging thrombolysis.

Overall, outcome did not differ between tPA+ASA and tPA-ASA patients. Outcome tended to be more favorable in tPA-ASA patients and was significantly better in antiplatelet naïve patients despite any differences in bleeding complications. The differences may be explained by a selection bias. Patients with prior antiplatelet therapy were older, had more comorbidities and more severe strokes mainly due to more ICA occlusions. The different etiopathogenesis of both groups reflects the treatment based on current guidelines where acute antiplatelet therapy is recommended for vessel-occlusions that require stenting in contrary to cardioembolic strokes. But when starting bridging thrombolysis the underlying stroke-etiology is not always obvious and the need for additional aspirin therapy in the acute phase just reveals during endovascular procedure. In a prospective trial the etiological differences should be considered in subgroup-analyses.

Given the lack of evidence on antiplatelet therapy during bridging thrombolysis, neurologists and interventional neuroradiologists have to balance the risk and benefit of additional aspirin administration during the intervention. In our study, bleeding complications in tPA+ASA patients were not increased. Given the often fatal consequences of proximal vessel reocclusions the benefit of antiplatelet monotherapy might outweigh the risk of intracerebral hemorrhage. Therefore, antiplatelet therapy should be considered when stenting of extra- or intracranial vessels has to be performed. However we cannot conclude from our data, that antiplatelets should routinely be provided in all patients after bridging thrombolysis.

The most important indication for administration of antiplatelets during endovascular procedures is the prevention of acute stent reocclusion when permanent stents have to be placed. Recent development of endovascular stroke treatment directs towards the use of retrievable stents where there is no indication for antiplatelets in general. But there are other indications for urgent administration of antiplatelets during endovascular interventions such as acute myocardial infarction (non STEMI) or severe atherosclerosis with high grade stenosis. Therefore, we believe that a trial assessing the safety of administration of antiplatelets during bridging thrombolysis is needed.

Our study has several limitations. First, this is a retrospective monocenter analysis of patients who underwent bridging thrombolysis with prospective follow-up assessment. Second, the number of patients and the number of bleeding complications was relatively low and the study period was limited from 11/2008 to end of 03/2014, just before the publication of the results of the major RCTs on EVT. Endovascular stroke therapy has a long tradition in our center and has been systematically performed in all patients with a proximal vessel occlusion in the anterior circulation, even before the publication of the RCTs. Therefore, the publication of the trials has not changed our treatment approach and including patients treated after the publication of the RCTs is unlikely to have influenced the results. Third, the reason for administration of aspirin during the intervention was mainly based on the decision of the treating physician and therefore a selection bias is possible. Fourth, the exact timing of Aspirin administration during bridging thrombolysis is not available in our database, making a time dependent analysis impossible. Fifth pharmacological intraarterial thrombolysis was performed with Urokinase. In our center, first endovascular stroke procedures were performed with Urokinase in 1992. After the publication of the PROACT II study, which proved that intraarterial thrombolysis with Pro-Urokinase is beneficial in patients with proximal middle cerebral artery occlusions we continued to treat patients with Urokinase as first line therapy for intraarterial thrombolysis. Regular publications of the Bernese Stroke Database showed promising results with no relevant increase in bleeding complications. Given our longstanding experience with Urokinase we had no convincing reason so far to change from Urokinase to intraarterial thrombolysis with rt-PA. However, our study does not give any information on bleeding complications in patients treated with aspirin in the setting of intraarterial administration of rt-PA.

Finally, we were not able to assess infarct volume at baseline and rupture of the blood brain barrier during follow-up from our dataset; both factors may have an important impact on hemorrhagic risk. But if hemorrhagic transformation was present on imaging, this was declared as an aICH and the rates of aICH did not differ significantly between both groups.

## Conclusions

In conclusion, antiplatelets therapy before or during bridging thrombolysis in severely affected patients with acute ischemic stroke due to large vessel occlusion did not increase the risk of bleeding complications and had no impact on outcome. However, our findings have to be confirmed in randomized controlled trials.

## References

[1] Zinkstok SM, Roos YB. Early administration of aspirin in patients treated with alteplase for acute ischaemic stroke: a randomised controlled trial. Lancet. 2012;380(9843):731–7. 10.1016/S0140-6736(12)60949-0 22748820

[2] Jauch EC, Saver JL, Adams HP Jr., Bruno A, Connors JJ, Demaerschalk BM, et al Guidelines for the early management of patients with acute ischemic stroke: a guideline for healthcare professionals from the American Heart Association/American Stroke Association. Stroke; a journal of cerebral circulation. 2013;44(3):870–947. 10.1161/STR.0b013e318284056a 23370205

[3] Guidelines for management of ischaemic stroke and transient ischaemic attack 2008. Cerebrovasc Dis. 2008;25(5):457–507. 10.1159/000131083 18477843

[4] www.clinicaltrials.gov. identifier NCT01657461.

[5] Broderick JP, Palesch YY, Demchuk AM, Yeatts SD, Khatri P, Hill MD, et al Endovascular therapy after intravenous t-PA versus t-PA alone for stroke. The New England journal of medicine. 2013;368(10):893–903. 10.1056/NEJMoa1214300 23390923 PMC3651875

[6] Mazighi M, Meseguer E, Labreuche J, Amarenco P. Bridging therapy in acute ischemic stroke: a systematic review and meta-analysis. Stroke; a journal of cerebral circulation. 2012;43(5):1302–8. 10.1161/STROKEAHA.111.635029 22529310

[7] Berkhemer OA, Fransen PS, Beumer D, van den Berg LA, Lingsma HF, Yoo AJ, et al A randomized trial of intraarterial treatment for acute ischemic stroke. The New England journal of medicine. 2015;372(1):11–20. 10.1056/NEJMoa1411587 25517348

[8] Nedeltchev K, Brekenfeld C, Remonda L, Ozdoba C, Do DD, Arnold M, et al Internal carotid artery stent implantation in 25 patients with acute stroke: preliminary results. Radiology. 2005;237(3):1029–37. 10.1148/radiol.2373041537 16237137

[9] Fischer U, Mono ML, Schroth G, Jung S, Mordasini P, El-Koussy M, et al Endovascular therapy in 201 patients with acute symptomatic occlusion of the internal carotid artery. European journal of neurology: the official journal of the European Federation of Neurological Societies. 2013;20(7):1017–24, e87. 10.1111/ene.12094 23398194

[10] Enomoto Y, Yoshimura S. Antiplatelet therapy for carotid artery stenting. Interventional neurology. 2013;1(3–4):151–63. 10.1159/000351686 25187775 PMC4031772

[11] Chaturvedi S, Yadav JS. The role of antiplatelet therapy in carotid stenting for ischemic stroke prevention. Stroke; a journal of cerebral circulation. 2006;37(6):1572–7. 10.1161/01.STR.0000221298.43117.be 16627791

[12] Kurre W, Perez MA, Horvath D, Schmid E, Bazner H, Henkes H. Does mechanical thrombectomy in acute embolic stroke have long-term side effects on intracranial vessels? An angiographic follow-up study. Cardiovascular and interventional radiology. 2013;36(3):629–36. 10.1007/s00270-012-0496-8 23086452

[13] Almekhlafi MA, Davalos A, Bonafe A, Chapot R, Gralla J, Pereira VM, et al Impact of age and baseline NIHSS scores on clinical outcomes in the mechanical thrombectomy using solitaire FR in acute ischemic stroke study. AJNR American journal of neuroradiology. 2014;35(7):1337–40. 10.3174/ajnr.A3855 24557701 PMC7966577

[14] Adams HP Jr., Bendixen BH, Kappelle LJ, Biller J, Love BB, Gordon DL, et al Classification of subtype of acute ischemic stroke. Definitions for use in a multicenter clinical trial. TOAST. Trial of Org 10172 in Acute Stroke Treatment. Stroke; a journal of cerebral circulation. 1993;24(1):35–41. 10.1161/01.str.24.1.35 7678184

[15] Heldner MR, Jung S, Zubler C, Mordasini P, Weck A, Mono ML, et al Outcome of patients with occlusions of the internal carotid artery or the main stem of the middle cerebral artery with NIHSS score of less than 5: comparison between thrombolysed and non-thrombolysed patients. Journal of neurology, neurosurgery, and psychiatry. 2014. 10.1136/jnnp-2014-308401 25266203

[16] Luedi R, Hsieh K, Slezak A, El-Koussy M, Fischer U, Heldner MR, et al Age dependency of safety and outcome of endovascular therapy for acute stroke. Journal of neurology. 2014;261(8):1622–7. 10.1007/s00415-014-7401-0 24916832

[17] Kase CS, Furlan AJ, Wechsler LR, Higashida RT, Rowley HA, Hart RG, et al Cerebral hemorrhage after intra-arterial thrombolysis for ischemic stroke: the PROACT II trial. Neurology. 2001;57(9):1603–10. 11706099 10.1212/wnl.57.9.1603

[18] The Thrombolysis in Myocardial Infarction (TIMI) trial. Phase I findings. TIMI Study Group. The New England journal of medicine. 1985;312(14):932–6. 4038784 10.1056/NEJM198504043121437

[19] Diener HC, Foerch C, Riess H, Rother J, Schroth G, Weber R. Treatment of acute ischaemic stroke with thrombolysis or thrombectomy in patients receiving anti-thrombotic treatment. Lancet neurology. 2013;12(7):677–88. 10.1016/S1474-4422(13)70101-7 23726849

[20] Diedler J, Ahmed N, Sykora M, Uyttenboogaart M, Overgaard K, Luijckx GJ, et al Safety of intravenous thrombolysis for acute ischemic stroke in patients receiving antiplatelet therapy at stroke onset. Stroke; a journal of cerebral circulation. 2010;41(2):288–94. 10.1161/STROKEAHA.109.559724 20056933

[21] Alexandrov AV, Grotta JC. Arterial reocclusion in stroke patients treated with intravenous tissue plasminogen activator. Neurology. 2002;59(6):862–7. 12297567 10.1212/wnl.59.6.862

[22] Rubiera M, Alvarez-Sabin J, Ribo M, Montaner J, Santamarina E, Arenillas JF, et al Predictors of early arterial reocclusion after tissue plasminogen activator-induced recanalization in acute ischemic stroke. Stroke; a journal of cerebral circulation. 2005;36(7):1452–6. 10.1161/01.STR.0000170711.43405.81 15947260

[23] Saqqur M, Molina CA, Salam A, Siddiqui M, Ribo M, Uchino K, et al Clinical deterioration after intravenous recombinant tissue plasminogen activator treatment: a multicenter transcranial Doppler study. Stroke; a journal of cerebral circulation. 2007;38(1):69–74. 10.1161/01.STR.0000251800.01964.f6 17138949

[24] Bates ER, Babb JD, Casey DE Jr., Cates CU, Duckwiler GR, Feldman TE, et al ACCF/SCAI/SVMB/SIR/ASITN 2007 Clinical Expert Consensus Document on carotid stenting. Vasc Med. 2007;12(1):35–83. 17451093 10.1177/1358863X06076103

[25] Mantese VA, Timaran CH, Chiu D, Begg RJ, Brott TG. The Carotid Revascularization Endarterectomy versus Stenting Trial (CREST): stenting versus carotid endarterectomy for carotid disease. Stroke; a journal of cerebral circulation. 2010;41(10 Suppl):S31–4. 10.1161/STROKEAHA.110.595330 PMC3058352 20876500

[26] Ederle J, Dobson J, Featherstone RL, Bonati LH, van der Worp HB, de Borst GJ, et al Carotid artery stenting compared with endarterectomy in patients with symptomatic carotid stenosis (International Carotid Stenting Study): an interim analysis of a randomised controlled trial. Lancet. 2010;375(9719):985–97. 10.1016/S0140-6736(10)60239-5 20189239 PMC2849002

[27] Mas JL, Arquizan C, Calvet D, Viguier A, Albucher JF, Piquet P, et al Long-term follow-up study of endarterectomy versus angioplasty in patients with symptomatic severe carotid stenosis trial. Stroke; a journal of cerebral circulation. 2014;45(9):2750–6. 10.1161/STROKEAHA.114.005671 25082808

[28] Ederle J, Bonati LH, Dobson J, Featherstone RL, Gaines PA, Beard JD, et al Endovascular treatment with angioplasty or stenting versus endarterectomy in patients with carotid artery stenosis in the Carotid and Vertebral Artery Transluminal Angioplasty Study (CAVATAS): long-term follow-up of a randomised trial. Lancet neurology. 2009;8(10):898–907. 10.1016/S1474-4422(09)70228-5 19717345 PMC2755037

[29] Mauri L, Kereiakes DJ, Yeh RW, Driscoll-Shempp P, Cutlip DE, Steg PG, et al Twelve or 30 Months of Dual Antiplatelet Therapy after Drug-Eluting Stents. The New England journal of medicine. 2014. 10.1056/NEJMoa1409312 PMC4481318 25399658

